# Supplementary material for: VariantStore: an index for large-scale genomic variant search
Source: Genome Biol. 2021 Aug 19;22:231. doi: 10.1186/s13059-021-02442-8 (PMC8375130; doi:10.1186/s13059-021-02442-8)
Supplement: Supplementary file 1 — Additional file 1 Review history [file 13059_2021_2442_MOESM1_ESM.docx]

Review History

**First round of review**

**Reviewer 1**

**Were you able to assess all statistics in the manuscript, including the appropriateness of statistical tests used?** Yes: No statistical tests are used in the manuscript.

**Were you able to directly test the methods?** No

**Comments to author:**

Pandey et al. present VariantStore, a tool written in C/C++. VariantStore answers variant queries over a large number of samples. Unique to VariantStore is its ability to answer queries using sample-specific coordinates. The manuscript focusses on computational aspects (index construction time, disk space, query throughput) and the effect of different kinds of queries and datasets on these computational aspects. I am not sure if Genome Biology is the most appropriate journal for this work. The paper is well-written and well-structured and is overall easy to read and understand. Even though I did not test the actual implementation myself, the Github page appears to contain sufficient documentation.

Major concerns:

-	The comparison with VB toolkit appears somewhat awkward as VG toolkit and VariantStore answer different types of queries: VG toolkit comprises a substring index to map reads against a variant graph whereas VariantStore retrieves sequences/variants based on input coordinates. As the underlying graph representations appear vastly different (both in data structure and in functionality) it is not immediately clear what is the benefit of the comparison in the "index construction" section of the paper.

-	The key functionality of VariantStore appears to be the ability to query variants in sample-specific coordinates (query 3 and 5). The authors should explain this aspect better since it is not immediately clear for this reviewer what is the added value over using reference coordinates. This becomes especially relevant when VariantStore is compared to existing reference-based tools like GTC: the latter is shown to be an order of magnitude faster. In its present form, the manuscript does not really allow readers to make an informed decision in terms of this functionality versus speed tradeoff.

**Reviewer 2**

**Were you able to assess all statistics in the manuscript, including the appropriateness of statistical tests used?** Yes

**Were you able to directly test the methods?** No

**Comments to author:**

The manuscript "VariantStore: An Index for Large-Scale Genomic Variant Search" by Pandey, Gao, and Kingsford describes a new computational method and software implementation for indexing large sets of variants with reference-based and sample-based coordinates. Being able to efficiently query sample-based coordinates in a scalable way is the scientific advance described here. The authors compare their method's efficiency in indexing characteristics and variant querying speed to similar previously published methods VG and GTC, respectively. The VariantStore index construction is shown to be constructed faster, use less disk space, and use less RAM than VG on one data set that VG was able to fully process. While VariantStore has querying capabilities for sample-based references, the implementation carries a cost such that reference-based queries are slower than the alternative GTC.

While the method is well described with relevant benchmarking efforts, the manuscript lacks a component that demonstrates how the new sample-based coordinate querying functionality would empower one to address some biological question of interest. This manuscript is almost entirely technical. From looking at the GitHub repo for VariantStore, it has the appearance of a proof-of-principle software implementation, in stark contrast to VG which exists as a tool targeted to supporting research activities. This further leads me to question exactly how users are intended to leverage VariantStore for biological research - which would be important for Genome Biology readership. For example, I could envision that there's a variant that is best described in sample-based coordinates for an important reason, and we need to identify all samples in the large data set that contain that variant. Pulling out that variant, the region around it, and drawing it in a graphviz dot format (as in VG) would be sufficiently compelling in my view.

Misc questions and comments:

Requiring RAM at 10% of the data size would seem to continue to be some barrier towards scalability. It might be worth commenting on other scalable approaches towards querying and analyzing variants, such as Hail: https://hail.is/docs/0.2/

Is it important that a consistent left-normalization approach be applied to the vcfs before you build an index?

The description of the TCGA data being used is confusing both in the abstract and in the Data section on page 6. In the abstract, it states TCGA-BRCA, but should just state TCGA, since BRCA is just one of the few different cancer types examined here. The counts of samples and files should be reexamined. BRCA, in particular, has ~1k samples yet there are ~4k vcf files available for download. These ~4k files (as shown on the GDC portal) include collections of vcfs and MAFs derived from different methods with multiple vcfs available per sample. If the goal is to address some biological question that's relevant to these targeted data sets, it would require careful review of the inputs being used here.

Figures 1, 2, and 6 would be better represented as line-point plots than bar plots.

**Authors Response**

**Point-by-point responses to the reviewers’ comments:**

**Reviewer 1:**

Pandey et al. present VariantStore, a tool written in C/C++. VariantStore answers variant queries over a large number of samples. Unique to VariantStore is its ability to answer queries using sample-specific coordinates. The manuscript focusses on computational aspects (index construction time, disk space, query throughput) and the effect of different kinds of queries and datasets on these computational aspects. I am not sure if Genome Biology is the most appropriate journal for this work. The paper is well-written and well-structured and is overall easy to read and understand. Even though I did not test the actual implementation myself, the Github page appears to contain sufficient documentation.

Major concerns:

-	The comparison with VB toolkit appears somewhat awkward as VG toolkit and VariantStore answer different types of queries: VG toolkit comprises a substring index to map reads against a variant graph whereas VariantStore retrieves sequences/variants based on input coordinates. As the underlying graph representations appear vastly different (both in data structure and in functionality) it is not immediately clear what is the benefit of the comparison in the "index construction" section of the paper.

*Response: We understand that VG toolkit is not designed for variant queries. However, similar to VariantStore, VG toolkit also stores variants in a variation graph and indexes the variants using succinct data structures to support multiple coordinate systems. One can repurpose the variation graph representation in the VG toolkit to answer variant queries. However, VG toolkit representation does not scale to thousands of samples. We include the VG toolkit in our evaluation to show the limitation of existing variation graph representations to scale to data containing thousands of samples. Moreover, given the efficiency of VariantStore, we argue that the variation graph representation in VariantStore can be repurposed to support string queries similar to VG toolkit.*

-	The key functionality of VariantStore appears to be the ability to query variants in sample-specific coordinates (query 3 and 5). The authors should explain this aspect better since it is not immediately clear for this reviewer what is the added value over using reference coordinates. This becomes especially relevant when VariantStore is compared to existing reference-based tools like GTC: the latter is shown to be an order of magnitude faster. In its present form, the manuscript does not really allow readers to make an informed decision in terms of this functionality versus speed tradeoff.

*Response: We have updated the introduction to include concrete examples to argue the need of variant queries based on sample specific coordinate systems. Following are the main points:*

*The main goal of pan-genomic variation analysis is to avoid biases that arise when treating a single genome as the reference. There is no single reference in pangenomes and any sample can be treated as a reference. In pan-genomic variation analysis, we can identify and study variants based on any sample genome and compare variants from two samples directly without the need for a reference genome.*

*The ability to treat any sample as a reference enables comparison of variants derived from different reference species. This is often required in analysis of plant genomes to compare variants in orthologs (a set of genes that have descended from a single common ancestor) across genomes from closely related species.*

*The ability to treat any sample as a reference enables access of variants using different versions of standard reference genomes. Having the ability to compare sample variants that are based on different reference genomes (e.g., GRCh37 and GRCh38) would make the process of adapting a new reference version less cumbersome.*

*Traditional reference-based representations have certain limitations. For example, storing highly variant sequence repertoire can be challenging because choosing a single representative sequence as reference is no longer viable and a bad choice of reference could lead to inefficient storage. For example, regions with high diversity, like T cell receptors (TCR), of which one person could have 10^13 different clonotypes, are stored in a multi-coordinate structure to preserve diversity rather than a single reference-based structure.*

**Reviewer 2:**

The manuscript "VariantStore: An Index for Large-Scale Genomic Variant Search" by Pandey, Gao, and Kingsford describes a new computational method and software implementation for indexing large sets of variants with reference-based and sample-based coordinates. Being able to efficiently query sample-based coordinates in a scalable way is the scientific advance described here. The authors compare their method's efficiency in indexing characteristics and variant querying speed to similar previously published methods VG and GTC, respectively. The VariantStore index construction is shown to be constructed faster, use less disk space, and use less RAM than VG on one data set that VG was able to fully process. While VariantStore has querying capabilities for sample-based references, the implementation carries a cost such that reference-based queries are slower than the alternative GTC.

While the method is well described with relevant benchmarking efforts, the manuscript lacks a component that demonstrates how the new sample-based coordinate querying functionality would empower one to address some biological question of interest.

*Response: We have updated our introduction to include concrete examples to argue for the need of variant queries based on sample specific coordinate systems. Following are the main points:*

*The main goal of pan-genomic variation analysis is to avoid biases that arise when treating a single genome as the reference. There is no single reference in pangenomes and any sample can be treated as a reference. In pan-genomic variation analysis, we can identify and study variants based on any sample genome and compare variants from two samples directly without the need for a reference genome.*

*The ability to treat any sample as a reference enables comparison of variants derived from different reference species. This is often required in analysis of plant genomes to compare variants in orthologs (a set of genes that have descended from a single common ancestor) across genomes from closely related species.*

*The ability to treat any sample as a reference enables access of variants using different versions of standard reference genomes. Having the ability to compare sample variants that are based on different reference genomes (e.g., GRCh37 and GRCh38) would make the process of adapting a new reference version less cumbersome.*

*Traditional reference-based representations have certain limitations. For example, storing highly variant sequence repertoire can be challenging because choosing a single representative sequence as reference is no longer viable and a bad choice of reference could lead to inefficient storage. For example, regions with high diversity, like T cell receptors (TCR), of which one person could have 10^13 different clonotypes, are stored in a multi-coordinate structure to preserve diversity rather than a single reference-based structure.*

This manuscript is almost entirely technical. From looking at the GitHub repo for VariantStore, it has the appearance of a proof-of-principle software implementation, in stark contrast to VG which exists as a tool targeted to supporting research activities. This further leads me to question exactly how users are intended to leverage VariantStore for biological research - which would be important for Genome Biology readership. For example, I could envision that there's a variant that is best described in sample-based coordinates for an important reason, and we need to identify all samples in the large data set that contain that variant. Pulling out that variant, the region around it, and drawing it in a graphviz dot format (as in VG) would be sufficiently compelling in my view.

*Response: The VartiantStore tool supports visualizing a local region of the variation graph. We have implemented an API based on Graphviz (dot) to visualize a subgraph containing all the nodes within a given number of hops from a variant node. This helps us look at the nearby region of the variation graph from a given variant. We implemented this API and also used it to visualize hot regions in the variation graph.*

*We have also added a CLI to output a subgraph in the dot format given a position in a sample name. Users can now directly use the CLI to visualize nearby variants in the variation graph. (https://github.com/Kingsford-Group/variantstore).*

Misc questions and comments:

Requiring RAM at 10% of the data size would seem to continue to be some barrier towards scalability. It might be worth commenting on other scalable approaches towards querying and analyzing variants, such as Hail: <https://hail.is/docs/0.2/>

*Response: The RAM usage is a configurable parameter and depends on the size of the node chunks in the partitioned variation graph representation. The size of the node chunks can be increased or decreased during construction. In our evaluation, we sized the node chunks to a few MBs to achieve good compression across node chunks and low memory usage.*

*Hail is a cloud-based solution for analyzing tabular and matrix data. It uses existing solutions from these cloud providers for indexing genomics data and performing queries. Hail’s infrastructure and API support in Python is complementary to the methodological advancement made by VariantStore. In future, we plan to extend VariantStore with a similar high-level API to build and query genomic variants.*

Is it important that a consistent left-normalization approach be applied to the vcfs before you build an index?

*Response:* *Good question. When storing variants from a new VCF file, VariantStore compares the REF with ALT and ignores the shared sequence from the left and only creates a branch starting from the first nucleotide that differs. Thus, VariantStore automatically does the left-normalization and it is okay if the original vcf is not left-normalized.*

The description of the TCGA data being used is confusing both in the abstract and in the Data section on page 6. In the abstract, it states TCGA-BRCA, but should just state TCGA, since BRCA is just one of the few different cancer types examined here. The counts of samples and files should be reexamined. BRCA, in particular, has ~1k samples yet there are ~4k vcf files available for download. These ~4k files (as shown on the GDC portal) include collections of vcfs and MAFs derived from different methods with multiple vcfs available per sample. If the goal is to address some biological question that's relevant to these targeted data sets, it would require careful review of the inputs being used here.

*Response: We have removed the BRCA from the abstract.*

*The TCGA-BRCA project contains 4319 VCF files (at the time of download; August 2019). Each VCF file contains two variant types; normal and tumor. In VariantStore, before inserting variants, we merge all the VCF files into one. During the merge process, we treat the two variant types in each file as separate samples. For the purposes of the evaluation, we report the number of samples as seen by VariantStore. We use the same data for constructing the VG toolkit representation.*

Figures 1, 2, and 6 would be better represented as line-point plots than bar plots.

*Response: We have added an overlay line plot in Figure 1, 2, and 6.*

**Second round of review**

**Reviewer 1**

No further questions.

**Reviewer 2**

I appreciate the authors' responses to my earlier comments and the corresponding revisions to the manuscript. However, I still continue to find the work more technical than I would normally expect for Genome Biology. I completely understand the value of graph genomes and the need to be able to align to and query across pan genome structures, and while there may be a need to query across different reference coordinate systems as opposed to a single reference coordinate system, the manuscript doesn't provide sufficient demonstration of this need. While the introduction is improved to define examples of where this may be needed, the actual demonstration within the manuscript continues to be insufficient. The example of using multiple references (hg37 vs hg38), while operationally interesting, still does little in my opinion to demonstrate the need for scaling to thousands of coordinate systems. Even in this case of hg37 vs hg38, I have serious doubts about the process used by the authors (involving minimap2, samtools, and freebayes) of providing an entirely faithful coordinate transformation between the two that one would rely on and obviate the need of doing variant calling on hg38 going forward. While technically VariantStore may be a scientific advance, for most GenomeBiology readers I suspect it would be considered a technical curiosity. If the authors could take examples provided in the introduction and make them tangible to would-be users, I would find it easier to advocate for GenomeBiology readership.
